# Automated modeling of polarons: defects and reactivity on TiO_2_(110) surfaces

**DOI:** 10.1038/s41524-026-01983-5

**Published:** 2026-04-09

**Authors:** Firat Yalcin, Carla Verdi, Viktor C. Birschitzky, Matthias Meier, Michael Wolloch, Michele Reticcioli

**Affiliations:** 1 University of Vienna, Faculty of Physics and Center for Computational Materials Science, Vienna, Austria; 2 School of Mathematics and Physics, The University of Queensland, Brisbane, QLD Australia; 3 VASP Software GmbH, Vienna, Austria; 4 National Research Council, CNR-SPIN, L’Aquila, Italy; 5 University of L’Aquila, L’Aquila, Italy; 6 TU Wien, Institute of Applied Physics, Vienna, Austria

**Keywords:** Materials science, Physics

## Abstract

Polarons are widespread in functional materials and are key to device performance in several technological applications. However, their effective impact on material behavior remains elusive, as condensed matter studies struggle to capture their intricate interplay with atomic defects in the crystal. In this work, we present an automated workflow for modeling polarons within density functional theory (DFT). Our approach enables a fully automatic identification of the most favorable polaronic configurations in the system. Machine learning techniques accelerate predictions, allowing for an efficient exploration of the defect-polaron configuration space. We apply this methodology to Nb-doped TiO_2_(110) surfaces, providing new insights into the role of defects in surface reactivity. Using CO adsorbates as a probe, we find that Nb doping has minimal impact on reactivity, whereas oxygen vacancies contribute significantly depending on their local arrangement via the stabilization of polarons on the surface atomic layer. Our package streamlines the modeling of charge trapping and polaron localization with high efficiency, enabling systematic, large-scale investigations of polaronic effects across complex material systems.

## Introduction

Polarizable materials can localize charge carriers into electronic states known as polarons^1–4^. In the limit of strong localization, the charge becomes confined almost entirely to a single lattice site, accompanied by distortions in the surrounding atomic structure^4^. Such “small polarons” play a critical role in determining the electronic, optical, and chemical properties of functional materials, with significant implications for technological applications^5–18^. The impact of polarons on material properties might vary depending on the localization site of the charge carrier^19–21^. For example, in catalysis, polarons on surface sites of the host material interact with molecular adsorbates, transferring electronic charge into the molecular orbitals and thereby acting as active centers; conversely, polarons populating subsurface layers tend to show no sizable effect on the adsorbates^22–25^. It is therefore crucial to carefully account for the polaron configuration space in order to provide an accurate description of polaronic materials.

Density functional theory (DFT) has been successfully used to model small polarons in a wide range of materials, especially transition metal oxides, providing insights on their fundamental properties in good agreement with experimental observations^13–15,26–31^. The supercell approach, where a small polaron is localized within a relatively large unit cell, allows us to inspect how polaron properties and effects depend on the particular charge localization sites, thus shedding light on the role of polarons in technological applications^21–23,31–39^. However, polaron modeling poses challenges to DFT simulations, especially when attempting to localize charge at custom (user-defined) lattice sites. In fact, instead of yielding the target polaronic state, in the DFT simulation the electronic charge might converge towards a delocalized-state solution (populating states in the conduction or valence bands rather than forming a polaronic state) or spontaneously localize on a different lattice site.

Several strategies have been proposed to facilitate control on polaron localization. These approaches typically involve multiple calculations to obtain the polaronic configuration at the target. An initial step is performed to obtain electronic density and atomic structure through charge constraints on specific electronic states, manual atomic distortions, or the addition of trapping potentials^40–43^. For example, computational tools such as the occupation matrix control (OCCMAT)^43^ allow the user to constrain electron localization on a specific site, in order to obtain a local atomic structure that effectively traps the charge carrier. Subsequently, the constraints are removed, and a standard DFT calculation is performed, using the constrained results of the first run as a starting point to fully relax electronic density and atomic structure. Careful manual intervention is required to monitor polaron localization in all calculation steps and to adopt corrective measures when localization fails (e.g., by imposing stricter constraints, resulting in more difficult and demanding convergence processes). These practical difficulties in modeling polaron localization may discourage careful inspections of the polaronic configuration space, leading to potentially spurious results in DFT studies of polaronic materials.

In addition to the intrinsic challenges of modeling polaron localization, the presence of atomic impurities such as point defects (e.g., vacancies, interstitials, substitutional dopants) further increases the complexity of simulations^44–46^: Defects and polarons interact through a complex interplay that proves intricate to fully unravel in computational modeling. On one hand, defects alter polaronic properties such as the polaron stability and the spatial distribution of the localized charge carriers in the material. In turn, polarons can impact the formation of defects and their arrangement in the material, potentially leading to structural phase transitions and drastic impacts on the material functionalities^20,32,46–53^. Understanding the diversity of properties and effects arising from the different defect-polaron arrangements requires careful exploration of the vast configuration space. Hence, the need for efficient and robust computational methods for polaron modeling.

**Fig. 1:**
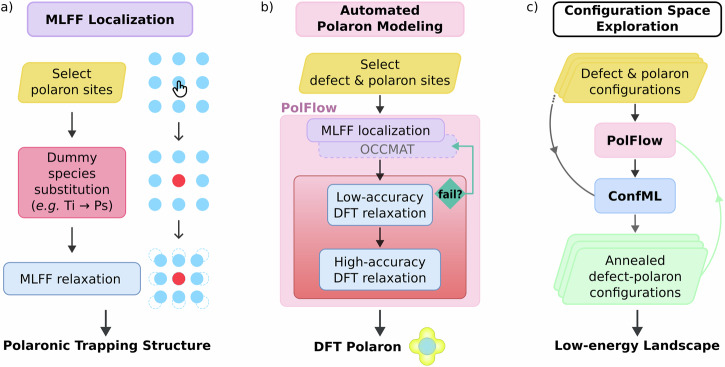
Sketch of the computational package components. **a** Polaron localization via the machine learning force field (MLFF) routine. A selected atomic site is labeled as polaronic site (Ps), and an MLFF-driven relaxation of internal forces is performed to obtain the polaronic trapping structure (see also Section “Localizing polarons via MLFF''). **b** Flowchart of the PolFlow workflow. The workflow can automatically perform a polaron localization calculation via the MLFF routine. In the case of a localization failure, stricter approaches are automatically adopted (e.g., the OCCMAT tool). PolFlow can progressively increase the accuracy of the calculations to achieve reliable DFT description of polaronic properties, saving computational time (see also Section “Workflow to streamline polaron modeling''). **c** The PolFlow workflow can automatically scan large numbers of defect-polaron configurations to explore the energy landscape. The interface with the configuration-space-exploration routine (ConfML) enables an active learning strategy to find low-energy configurations via simulated annealing (see also Section “Active learning for defect-polaron configurations'').

Here, we present a fully automated approach for polaron localization that can be used to efficiently sample the defect-polaron configuration space. As sketched in Fig. 1, our package includes a high-throughput workflow, PolFlow, that streamlines all steps required to obtain polaron localization and automatically monitors the status of the simulations in real time. In the case of localization failure, the calculation is immediately interrupted, a different localization strategy is adopted, and the calculation is resumed, with no manual intervention required. Furthermore, by leveraging machine learning (ML) force fields (MLFFs), we introduce a novel approach to polaron localization: we show that MLFFs can be adapted for polaron modeling, enabling an accurate description of the ground-state atomic structure in polaronic materials at a fraction of the computational cost of DFT calculations.

The software package featuring the high-throughput workflow PolFlow, together with the MLFF-powered localization technique, accelerates the calculations of small polarons and provides broader accessibility to users with limited expertise in polaron modeling. Furthermore, its high efficiency enables careful investigations of the polaronic configuration space^54,55^, as we complemented our workflow with an interface to the active-learning scheme ConfML^20^, to sample the large variety of defect and polaron configurations in materials.

We demonstrate the capabilities of our computational machinery by investigating the polaronic properties on the rutile TiO_2_(110) surface, modeling the simultaneous presence of intrinsic oxygen-vacancy (*V*_O_) defects and Nb doping atoms^56,57^. TiO_2_ is a prototypical polaronic material: In its pristine form, it is a wide-bandgap semiconductor with no in-gap states, yet it readily hosts polarons upon photoexcitation^5^ and/or in the presence of defects or doping^58^. Oxygen vacancies, which form particularly easily on the rutile (110) surface up to a concentration of ~17%^49,50^, donate two excess electrons that localize as two polarons. Likewise, common dopants typically included in the samples to tune electronic and catalytic properties can introduce additional carriers that also form polarons (e.g., one polaron per Nb dopant)^26,59–62^. Modeling the complex interplay between these atomic impurities and polarons is essential for understanding the properties of TiO_2_. In real samples, such impurities often coexist, yet treating them simultaneously within conventional computational frameworks is practically challenging due to the large configurational space. Nevertheless, their concurrent presence is likely to affect polaron properties in oxides in non-trivial ways^63–66^. The efficient PolFlow exploration allows us to understand key aspects of the most stable defect-polaron configurations on the surface and sets the basis for analyzing the role of polarons in surface reactivity, which we investigate here by using CO as a probe molecule. Our extensive sampling of the configuration space reveals that Nb dopants have a negligible direct impact on the reactivity and the stabilization of polaronic states. Oxygen vacancies, instead, play a major role, as specific *V*_O_ arrangements can promote the localization of polarons on the surface layer and their interaction with CO adsorbates.

This paper is organized as follows. In Section “Methods”, we present our automated approach to polaron localization and configuration space exploration, including the description of the PolFlow high-throughput workflow, the MLFF-powered localization technique, and the ConfML-powered configuration search. In Section “Results”, we apply our methodology to study the distribution of polarons, Nb dopants and *V*_O_ defects on TiO_2_(110) surfaces and analyze its performance. In Section “Discussion” we conclude by showing how the defect-polaron distribution, accessible through our efficient computational approach, influences technologically relevant properties such as CO adsorption on the surface.

## Results

We have developed a framework for automating the modeling of polarons (described in Section “Methods”). As sketched in Fig. 1, our package integrates three components: a machine-learning force-field (MLFF) localization module to model polaron trapping on selected target sites (Section “Localizing polarons via MLFF”); an efficient workflow (PolFlow) that drives the DFT simulations and automatically monitors status and convergence (Section “Workflow to streamline polaron modeling”); and an interface to an active learning engine (ConfML) that enables systematic exploration of the configuration space associated with polarons and atomic defects (Section “Active learning for defect-polaron configurations”).

**Fig. 2:**
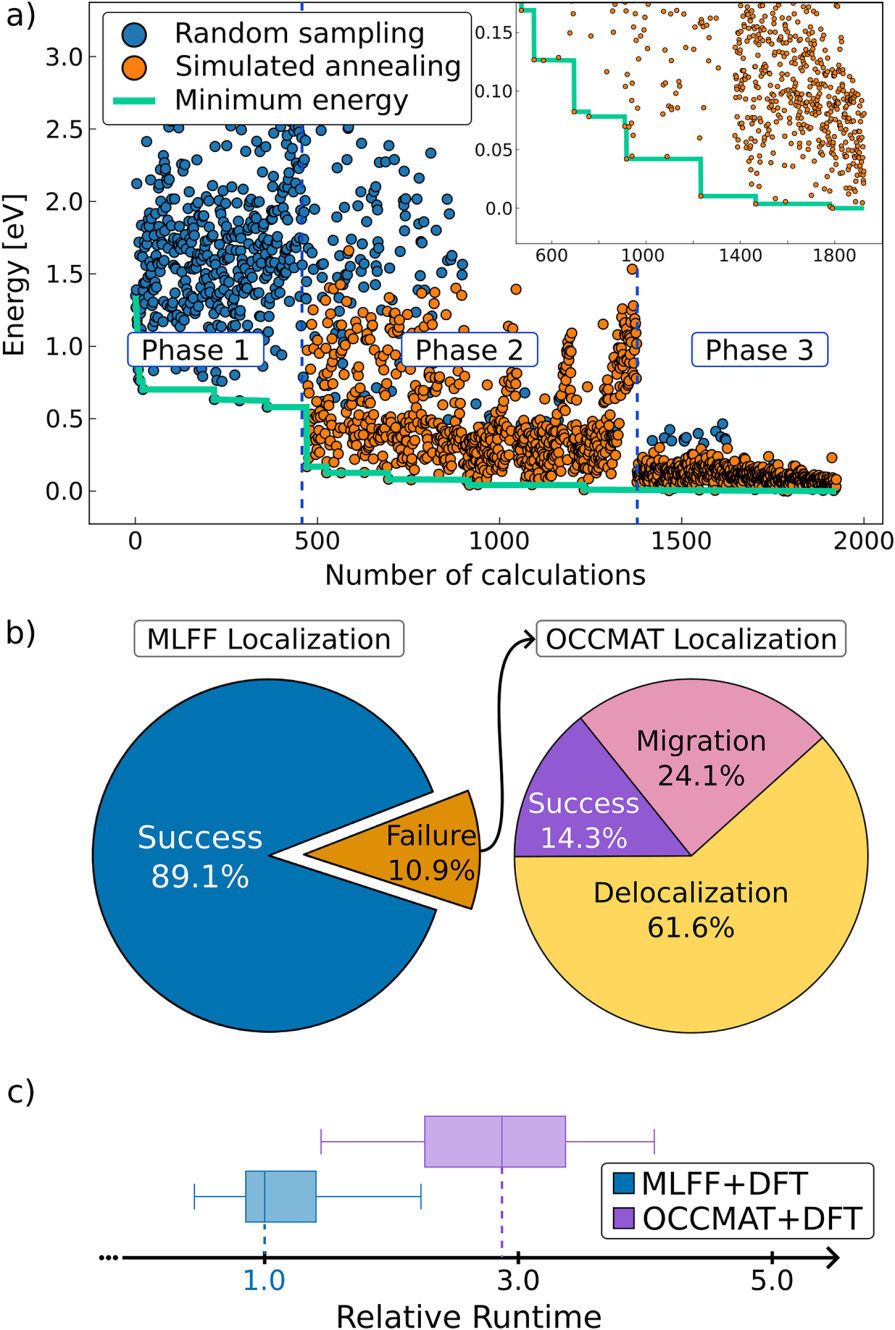
Performance analysis of our computational package in the configuration space exploration. **a** Energy landscape obtained via PolFlow, using random sampling and simulated annealing. The inset focuses on the low-energy configurations in Phases 2 and 3 (up to 0.15 eV). **b** Analysis of polaron localization outcomes using MLFF+DFT (left) and subsequent recovery attempts via OCCMAT (right). **c** Runtime of the MLFF+DFT and OCCMAT approaches in the low-accuracy runs (boxplots show the distribution of computational times normalized to the MLFF+DFT median).

**Fig. 3:**
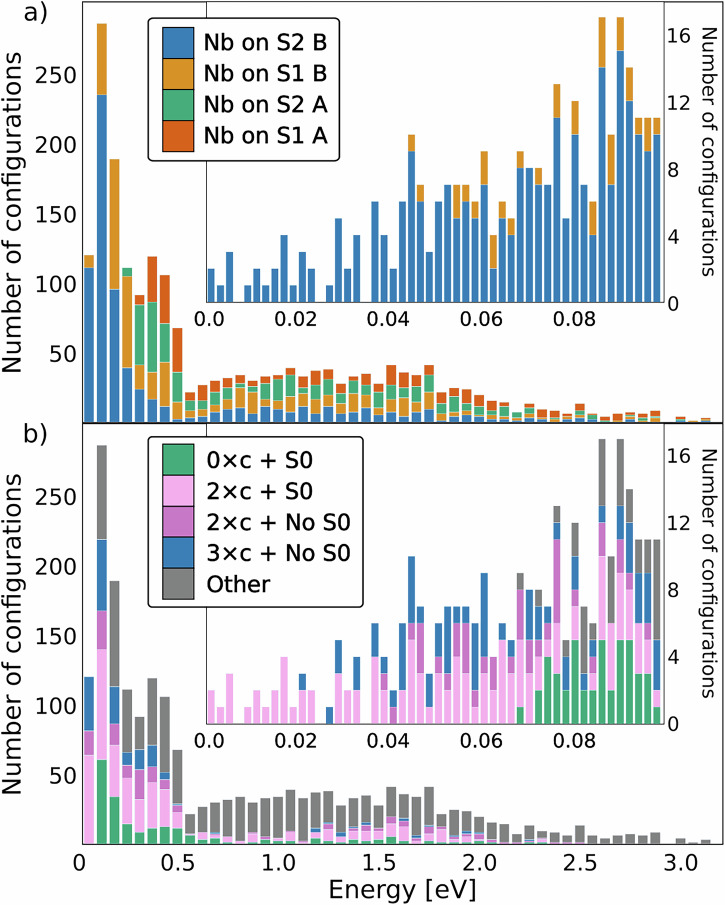
Distribution of configuration energies. **a** Histograms are colored according to the Nb position (A or B sites, on the S1 and S2 layers). **b** Colored histograms represent selected *V*_O_-polaron configurations (0 × c, 2 × c, 3 × c with/without surface polarons). Insets in both panels zoom in on the low-energy portion of the configuration space.

**Fig. 4:**
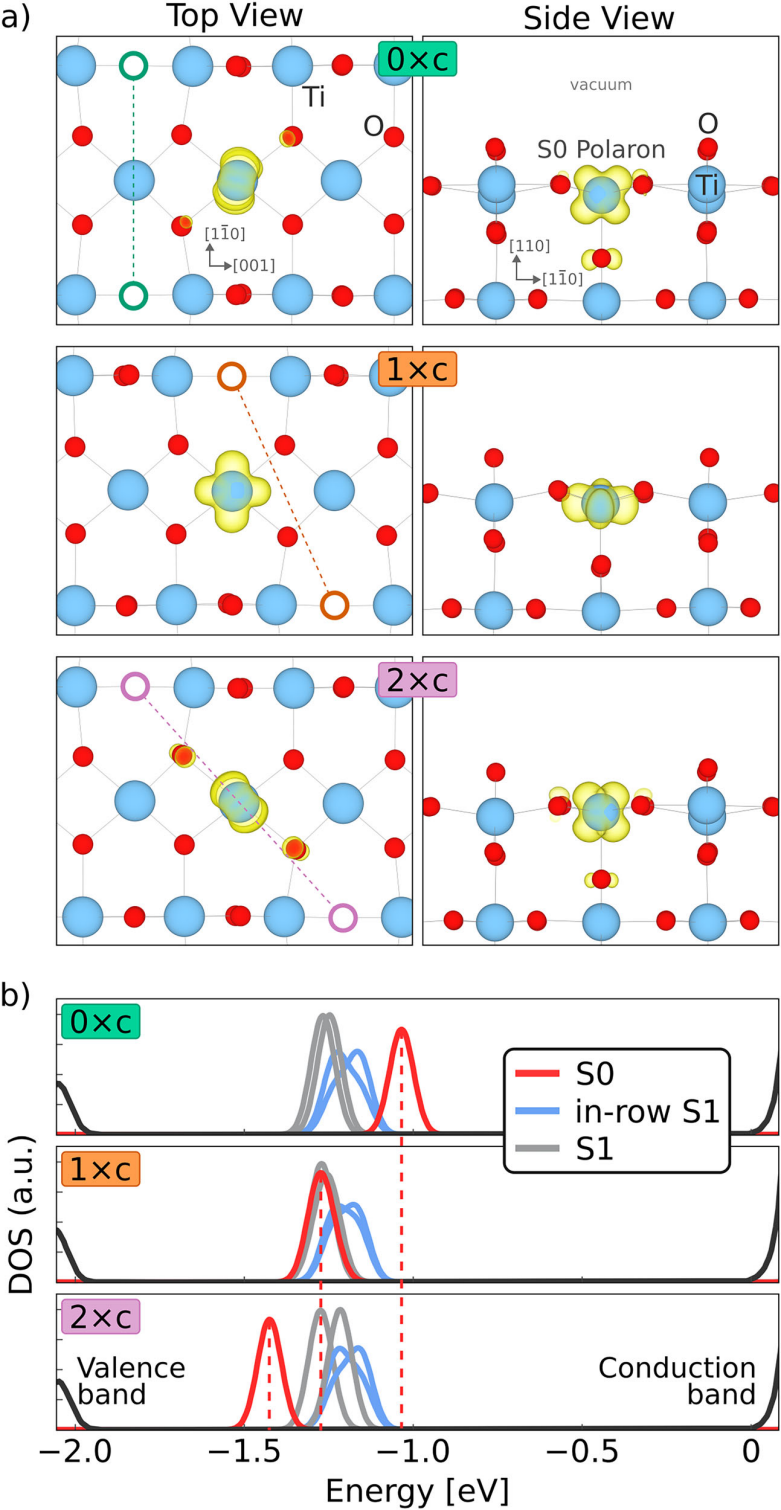
Oxygen-vacancies arrangements promoting surface polarons. **a** Top and side views of S0 polarons in the 0 × c, 1 × c, and 2 × c arrangements (circles indicate *V*_O_ sites). **b** Corresponding DOS, projected on S0 polarons (red) and S1 polarons either isolated (gray) or localizing three lattice sites away from a second polaron along the same [001] Ti row (blue); details of the DOS projection are shown in the atomic structures in Figure SF3. Dashed lines highlight the shift of the S0 polaron eigenstate due to the different *V*_O_ arrangements.

**Fig. 5:**
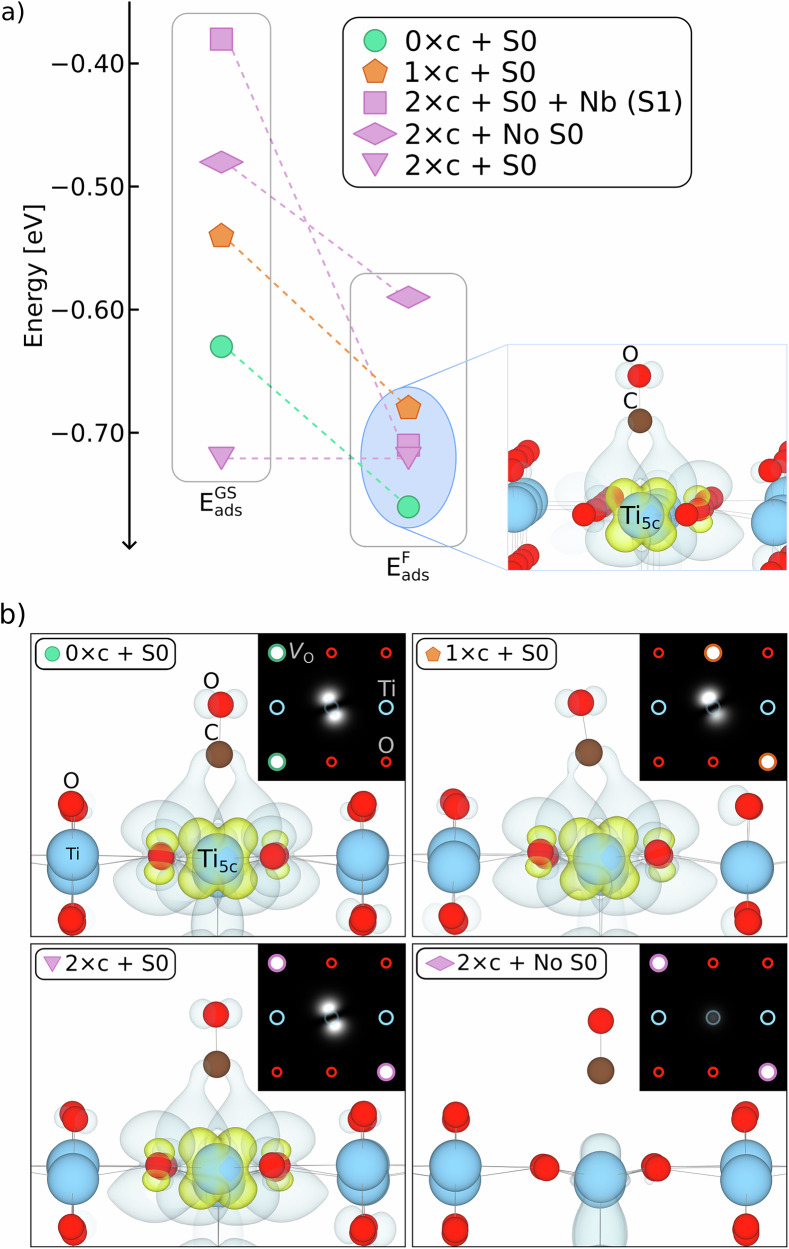
CO adsorption on T*i*_5c_ sites. **a**${E}_{{\rm{ads}}}^{GS}$ (left) and ${E}_{{\rm{ads}}}^{F}$ (right) energies for a CO molecule adsorbing on a T*i*_5c_ site hosting a surface S0 polaron in the proximity of two *V*_O_ defects in the 0 × c, 1 × c and 2 × c arrangements (circle, pentagon, and triangle symbols, respectively), with the Nb dopant on the S2 layer. For the 2 × c pattern, we also show the case of no surface polaron (diamond) and the case of Nb on the subsurface S1 layer (square). The inset shows the complex forming between CO and the S0 polaron (charge densities with low and high isovalues in light blue and yellow, respectively). **b** Charge density of selected cases from (**a**). Insets show the simulated STM images of the polaronic density (filled circles indicate the position of *V*_O_ defects).

This machinery allows us to efficiently study the most stable defect-polaron configurations of oxygen vacancies and Nb dopants on the TiO_2_(110) surface. In Section “Benchmark and performance analysis”, we comment on the performance of our approach (Fig. 2); in Section “Distribution of Nb dopants, VO defects, and polarons on TiO_2_(110)”, we focus on the physical outcomes of our test-case study (Figs. 3 and 4). The implications for surface reactivity are instead presented in Section “Discussion” (Fig. 5).

### Benchmark and performance analysis

Our goal is to assess and analyze the most stable defect-polaron configurations on or near the TiO_2_(110) surface. To this aim, we trained the ConfML search tool via the active learning scheme as implemented in the PolFlow package, adopting a multi-phase approach (see Fig. 2).

**Fig. 6:**
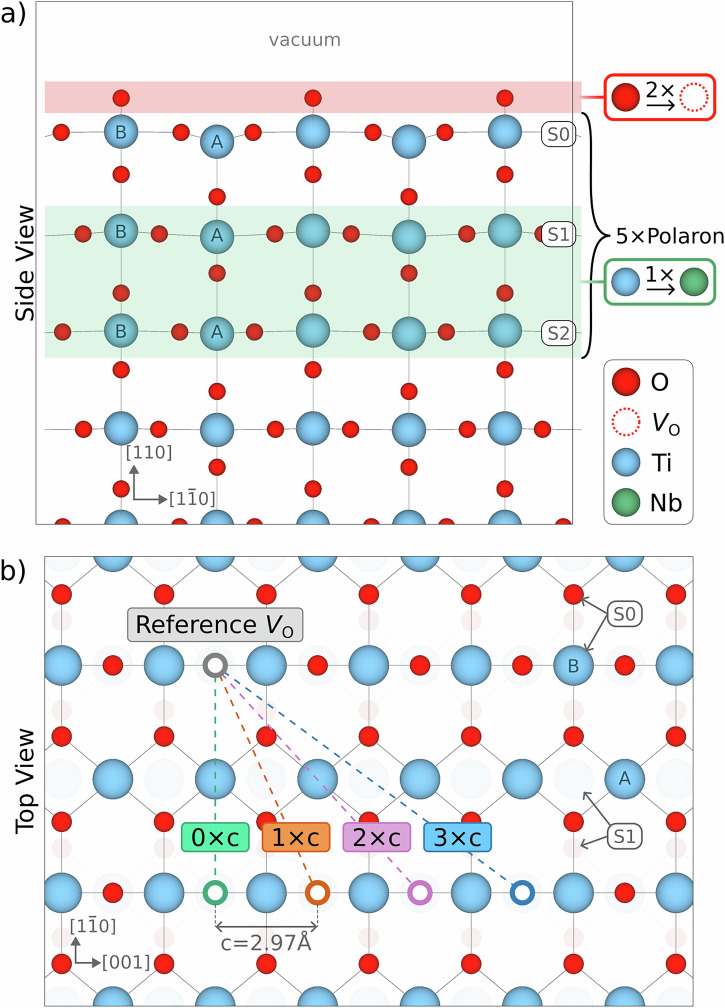
Structural model of TiO_2_(110). **a**, **b** shows the side and top views, respectively. Two of the surface oxygen-bridging atoms are removed to form *V*_O_ defects (see red box). Arrangements with the two oxygen vacancies on adjacent [001] O rows are labeled as 0 × c, 1 × c, 2 × c, 3 × c, which indicate the ideal distance from a reference *V*_O_ along the *c* axis. Polarons can localize on Ti A sites on the surface S0 layer, and on all (A and B) Ti sites of S1 and S2 layers. The green box indicates the Ti atoms on S1 and S2 layers that can be substituted by the Nb dopant.

Initially, in Phase 1, we modeled 198 slabs of TiO_2_(110), constructing 6 × 3 supercells with distinct arrangements of two *V*_O_ defects and one Nb dopant as described in Section “Computational setup for the TiO_2_(110) study case” (see also Fig. 6). For each slab, we generated three random polaron configurations, resulting in approximately 600 total defect-polaron configurations. Using PolFlow, we calculated at the DFT level the energy of all these configurations, taking advantage of the high efficiency of the MLFF-based localization method. Once we obtained a sufficiently large dataset (approximately 450 valid samples), we used it to train the ConfML model.

Next, in Phase 2, the ConfML model was iteratively refined by adopting our active learning approach on a mixture of random configurations and annealed samples proposed by the ML model itself. Finally, in Phase 3, we continued the active-learning training by including only the lowest-energy configurations that we progressively obtained in the annealing. This strategy allowed us to explore a large number of low-energy configurations [see inset in Fig. 2a].

The three-phase active-learning approach implemented in the PolFlow package enabled efficient training of ConfML on a database including in total 1924 symmetrically distinct configurations. The resulting ML model can predict the energy of any defect-polaron configuration on TiO_2_(110) with a mean absolute error (MAE) of 66 meV for our 6 × 3 slab, corresponding to approximately 13 meV per polaron (see Fig. SF1 in the Supplementary Information). The accuracy is further improved at low energies due to the annealing-based training in Phase 3 that prioritizes the most stable defect-polaron configurations. In fact, by considering only low-energy configurations (within 0.15 eV from the minimum, rather than the full range spanning 3 eV), the error is remarkably reduced to 32 meV for the entire slab (6 meV per polaron).

Figure 2b, c highlights the efficiency of the PolFlow package. In most cases (89%), it has been possible to successfully model defect-polaron configurations via an initial MLFF run to localize the polaron at user-defined positions, followed by DFT calculations to refine energy and atomic distortions (i.e., the MLFF + DFT approach). In the remaining 11% of cases, the localization of the polaron failed during the follow-up DFT run [left panel in Fig. 2b]. In response, the PolFlow package automatically employed the occupation matrix control tool to fix the localization failures: 14% of these OCCMAT calculations resulted in successful localization, while 24% led to polaron localization on different sites and 62% to delocalized excess [right panel in Fig. 2b]. Localization failures ought to be expected for some of the sites designed to host polarons, as some arrangements are particularly unstable: For example, adjacent Ti sites in the TiO_2_(110) subsurface typically do not host polarons unless a defect is present in the proximity^39^. In the cases of re-localization to different sites, if the new configuration is not symmetrically equivalent to any existing entry in the dataset, PolFlow continues the calculation and adds the configuration to the database. Conversely, if the resulting configuration is symmetrically equivalent to an existing sample, or in the case of electron delocalization, the workflow interrupts the calculation, saving computation time with no need for user intervention.

As shown in Fig. 2c, polaron localization using MLFF is significantly more efficient than alternative approaches. In our system, low-accuracy polaron localization via the OCCMAT + DFT approach required on average 3 times the computing time compared to the MLFF + DFT strategy (corresponding to 4.4 and 1.5 h per defect-polaron configuration, respectively, when using 64 CPU-cores on our machines). This gain in efficiency allowed us to save about one-third of the total computational cost in the overall (low- and high-accuracy) polaron-localization process during the exploration of the defect-polaron configuration space in TiO_2_(110) (saving in total approximately 300, 000 core hours on our 64-core machines for calculating 1924 symmetrically distinct configurations, see also Supplementary Fig. SF2). Moreover, we estimate that the PolFlow checks on failed polaron localizations (11% of the calculations in the MLFF+DFT, and 86% in the OCCMAT+DFT approaches) saved an additional 150, 000 core hours, along with a considerable amount of user time otherwise spent on setup and analysis of localization outcomes. All considered, the overall computing-time saving amounts to a reduction of nearly 50% in the total computational cost.

Summing up our benchmark analysis, we outline the main advantages of our integrated approach, which combines the MLFF polaron localization routine, the PolFlow automated workflow, and the ConfML interface for configuration space exploration. The MLFF method reduces computational time as compared to state-of-the-art approaches, while maintaining high reliability in polaron localization. Only 14% of the few failed MLFF localization runs were successfully corrected by the OCCMAT approach. For studies focused on low-energy structures, we find that skipping OCCMAT for failed MLFF cases is generally safe, as these typically correspond to unfavorable configurations, thus resulting in additional overall time saving. The automated PolFlow workflow and the ConfML interface together enable effortless configuration space exploration, even for users with limited experience in polaron modeling. Their use simplifies the setup and execution of simulations on highly complex systems with entangled interplay between multiple impurities, such as the present case involving polarons, Nb dopants, and oxygen vacancies. Overall, our computational framework provides a powerful tool for investigating key properties of polaronic materials, as showcased in the following section.

### Distribution of Nb dopants, *V*_O_ defects, and polarons on TiO_2_(110)

The extensive exploration of the Nb-*V*_O_-polaron configuration space described above allows us to identify the most stable defect arrangements on TiO_2_(110). Figure 3 collects our statistical analysis projected onto the Nb and *V*_O_ contributions (panels (a) and (b), respectively). The corresponding structural models and polaronic charge densities are shown in Fig. 4; see also Fig. 6 for a description of the nomenclature used to label the defect configurations.

Figure 3a reveals clear trends for Nb dopants. The B sites of TiO_2_(110) are more favorable substitutional sites compared to the A sites, which are at best 250 meV less stable. Moreover, there is a clear tendency for Nb to lie on the second subsurface layer: the inset in panel (a) shows a large number of low-energy S2 configurations, with the most stable one being almost 50 meV more favorable than the best S1 configuration. We note that the energy instability of Nb in the S1 layer seems to be associated with exceptionally high eigenvalues for S1 polarons in the proximity of the dopant, likely due to unfavorable trapping environment around the defect (see the density of states (DOS) in Fig. SF5 in the Supplementary Information).

Moving our attention to the *V*_O_ defects, Fig. 3b highlights a remarkable stability for the 2 × c arrangement. All of the most stable configurations up to 20 meV (see the inset) are formed by a surface S0 polaron in between two 2 × c oxygen vacancies [see the model in Fig. 4a]. This result for the 2 × c pattern, obtained here for a *V*_O_ concentration of 11% and Nb concentration of 0.5%, is in agreement with a previous computational study with different levels of impurities (*c*_V O_ = 17% and *c*_Nb_ = 0%) and with experimental scanning probe images of these patterns in various conditions^20,22^.

Conversely, the absence of surface polarons favors homogeneous distributions of oxygen vacancies (the 3 × c configurations) that are at best +20 meV less stable than the ground state (whereas 2 × c configurations with no surface polarons appear at higher energies, starting from +40 meV). Considering the lack of such homogeneous patterns in the experimental samples, this result corroborates the hypothesis that polarons play a key role in the distribution of *V*_O_ defects during the annealing process^20^: Surface S0 polarons likely mediate the vacancy-vacancy repulsion and promote the stabilization of 2 × c arrangements rather than the homogeneous distribution (which would be expected as dominant pattern arising from a purely repulsive *V*_O_-*V*_O_ interaction in the absence of intermediate polarons).

Configurations with other oxygen-vacancy arrangements, such as the 0 × c and 1 × c, appear only at higher energies (above +70 meV). Similar to the 2 × c ground state, also the 0 × c and 1 × c configurations promote the stabilization of surface polarons. As shown in Fig. 4, the surface polarons exhibit different orbital symmetries depending on the local oxygen-vacancy environment, as evident from the different orientations of the orbital densities in the three panels^31^. Moreover, the local *V*_O_ environment strongly affects the polaron stability: The DOS reported in Fig. 4 clearly shows a shift of the S0 polaron eigenvalue, depending on the *V*_O_ arrangement. We also note a broadening of the polaronic peaks in the DOS due to the repulsive interaction with other nearby polarons localizing in the same [001] Ti S1 row, three-lattice-site apart (blue curves, labeled as “in-row” in Fig. 4, also see Fig. SF4 in the Supplementary Information for a structural model of 2 × c), while isolated polarons show sharper eigenstates (gray curves). The repulsion between polarons on the same Ti row is indeed known to have a strong effect on the stability of the TiO_2_(110) surface itself, leading to reconstruction of the atomic structure at highly reducing conditions^49,50,67–72^. Oxygen vacancies in the proximity might mitigate the polaron-polaron repulsion, reducing the broadening of the polaronic peaks^39^.

In contrast to the main role played by *V*_O_ defects, our configuration space exploration suggests that Nb dopants do not play a primary role in the stabilization of surface polarons, as long as the dopant lies in the S2 layer. To verify this finding, we performed additional calculations: By taking advantage of our efficient PolFlow framework, we explored the polaronic landscape on TiO_2_(110) surfaces without Nb defects. In Table 1 we compare the polaron formation energy (*E*_POL_) of different configurations. The energy *E*_POL_ of subsurface polarons is obtained by comparing the total energy of the polaronic system (*E*^loc^) with respect to a solution without polaronic distortions and all electrons delocalized (*E*^del^):

1 $${E}_{{\rm{POL}}}(S1)=\left({E}^{{\rm{loc}}}-{E}^{{\rm{del}}}\right)/{n}_{{\rm{S1}}}$$

where *n*_S1_ is the number of subsurface polarons. In the absence of surface polarons, the optimal 2 × c configuration with Nb on the S2 layer yields an average *E*_POL_ energy of −450 meV per S1 polaron. Assuming this energy value for S1 polarons unchanged, we can evaluate the formation energy for the S0 polaron using the following expression:

2 $${E}_{{\rm{POL}}}(S0)={E}^{{\rm{loc}}}-{E}^{{\rm{del}}}-{n}_{{\rm{S1}}}\times {E}_{{\rm{POL}}}(S1)\,.$$

We obtain *E*_POL_(S0) = −490 meV in the 2 × c configuration with Nb, thus an energy gain *ΔE*_POL_ of −40 meV upon promotion of a S1 polaron to the surface.

**Table 1:** Polaron formation energies for surface and subsurface polarons, in the presence and absence of Nb dopants (labeled as “Nb on S2” and “no Nb”, respectively)

Polaron formation	Nb on S2	no Nb
*E*_POL_(S0)	−490 meV	−530 meV
*E*_POL_(S1)	−450 meV	−445 meV
Δ*E*_POL_(S0–S1)	−40 meV	−85 meV

The oxygen vacancies are in the 2 × c pattern. All polarons have been annealed via PolFlow, and the best configurations with and without surface polarons are considered in the Table.

By removing the Nb defect and annealing via PolFlow the polarons in the 2 × c configuration, we can calculate the formation energies for surface polarons in the absence of extrinsic dopants: *E*_POL_(S1) = −445 meV (similar to the Nb case reported above), and *E*_POL_(S0) = −530 meV. As evident, the energy gain of a polaron localizing on the surface compared to the subsurface increases to Δ*E*_POL_ = −85 meV, practically doubling the value obtained in the Nb-doped case. From this analysis, we conclude that Nb dopants do show an attractive interaction with polarons: The Nb impurity lying on the S2 layer worsens the stability of the S0 polaron by 45 meV. However, this effect is relatively minor when compared to the *E*_POL_ energy of the surface polaron in the 2 × c complex, which is an order of magnitude larger. Thus, Nb does not ultimately prevent the stabilization of surface polarons.

As such, doping rutile TiO_2_ with Nb has been found to be an effective method for introducing additional electrons into the system without altering the surface structure. By enabling a systematic exploration and simultaneous treatment of vacancies and dopants, our efficient approach clearly shows that oxygen vacancies drive polaron stabilization on the rutile surface, whereas Nb serves mainly as a source of additional charge carriers and is practically unable to alter the optimal *V*_O_− induced patterns (in fact, previous studies modeling exclusively oxygen vacancies in rutile without dopants predict similar low-energy arrangements)^20,49,54,73–75^. This property is particularly advantageous for imaging purposes, such as scanning tunneling microscopy, as it enables conductivity in the bulk, while the preservation of the surface structure might be crucial for applications such as catalysis, where active sites for adsorption and catalytic reactions remain intact.

## Discussion

Clarifying the distribution of defects and polarons is key to determining the properties of materials and advancing technological applications. Here, we use the results of our extensive PolFlow characterization study to shed light on crucial aspects of TiO_2_(110) surface reactivity, by modeling CO adsorption on the undercoordinated Ti sites (T*i*_5c_)^21–24,76–87^.

Figure 5 collects our analysis for the most favorable polaron-Nb arrangements obtained through the PolFlow-ConfML exploration for the *V*_O_ patterns 0 × c, 1 × c, and 2 × c. These patterns favor the formation of a polaron on the surface layer, in the proximity of the oxygen vacancies (see Fig. 4). Additionally, for the 2 × c arrangement, we also considered the most favorable configuration featuring no surface polaron (all five polarons are on the S1 subsurface layer, labeled by a diamond symbol). Nb is on the S2 subsubsurface layer, except for the configuration labeled by a square symbol, where Nb lies on an S1 site below the S0 polaron (see also model structure in Fig. SF5). We modeled CO adsorbing directly above the T*i*_5c_ site hosting the surface polaron (or in the corresponding site in between the two *V*_O_s for the diamond case with all S1 polarons).

For every defect-polaron configuration *x* analyzed, we computed two distinct adsorption energies, ${E}_{{\rm{ads}}}^{GS}$ and ${E}_{{\rm{ads}}}^{F}$, to inspect different contributions to the adsorption of CO molecules:

3 $$\begin{array}{rcl}{E}_{{\rm{ads}}}^{{\rm{GS}}}(x) & = & {E}_{{\rm{slab+CO}}}(x)-({E}_{{\rm{slab}}}(GS)+{E}_{{\rm{CO}}})\,,\\ {E}_{{\rm{ads}}}^{{\rm{F}}}(x) & = & {E}_{{\rm{slab+CO}}}(x)-({E}_{{\rm{slab}}}(x)+{E}_{{\rm{CO}}})\,.\end{array}$$

Here, *E*_slab+CO_(*x*) is the energy of TiO_2_(110) with an adsorbed CO molecule in the defect-polaron configuration *x*; *E*_slab_ is the energy of the TiO_2_(110) surface with no adsorbate (referred to as bare surface), modeled either in its ground-state defect-polaron configuration [*E*_slab_(*GS*), see GS model structure in Fig. SF4] or in the defect-polaron configuration *x* [*E*_slab_(*x*), i.e., both the bare surface and the slab with the adsorbate are modeled in the configuration *x*]; and *E*_CO_ is the energy of a CO molecule in the gas phase. We note that, in all cases, the ionic positions were relaxed to minimize the internal forces, as described in Section “Methods”. These quantities allow us to determine the overall most stable adsorption scenario through ${E}_{{\rm{ads}}}^{GS}$ (since the ground state of the bare surface is used as reference for the adsorption in any configuration *x*), as well as the direct contribution of the polarons to the adsorption via ${E}_{{\rm{ads}}}^{F}$(in fact, fixing the defect-polaron configuration in the absorption process removes any re-configurational contribution to the energy).

In Fig. 5a, the left column shows the ${E}_{{\rm{ads}}}^{GS}$ values calculated for all the different defect distributions under consideration. The most favorable CO adsorption scenario is given by the 2 × c arrangement, with the molecule adsorbing on a surface polaron trapping in between the two oxygen vacancies (represented by a triangle symbol). This result is in line with previous DFT calculations modeling selected *V*_O_ configurations on the Nb-free TiO_2_(110) surface^22^, thus suggesting a minor role of Nb dopants in CO adsorption (see also discussion on the effects of Nb at the end of this Section).

The adsorption becomes progressively less favorable for the polaron-CO complexes at the 0 × c and 1 × c *V*_O_ patterns [see ${E}_{{\rm{ads}}}^{GS}$ for the circle and pentagon symbols in Fig. 5a], and even worse for configurations with no surface polaron (see the diamond symbol, representing the 2 × c configuration with all polarons in the subsurface layer). Finally, the configuration with an Nb impurity on the subsurface layer S1 right below the adsorption site yields the most unfavorable ${E}_{{\rm{ads}}}^{GS}$ energy (square symbol). This behavior originates from the intrinsic instability of configurations with shallow Nb defects, rather than from the interaction of the dopant with CO itself, as discussed in the analysis of the ${E}_{{\rm{ads}}}^{F}$ energy below.

The ${E}_{{\rm{ads}}}^{F}$ energy [right column in Fig. 5a] clarifies the role of surface polarons in stabilizing the CO adsorption, and the minor contribution of Nb. In fact, in the expression for ${E}_{{\rm{ads}}}^{F}\,(x)$, the reference energy *E*_slab_(*x*) is obtained from a system with no CO adsorbate, while defects and polarons are kept at the same sites as in the slab adsorbing the molecule; therefore, at variance with ${E}_{{\rm{ads}}}^{GS}$, the adsorption energy ${E}_{{\rm{ads}}}^{F}$ is not affected by the configuration energy of defects and polarons, and allows us to inspect the direct contributions of local impurities to CO adsorption. As evident from the right column in Fig. 5a, a CO molecule adsorbing on a surface polaron shows very similar ${E}_{{\rm{ads}}}^{F}$ energies (within −760 meV and −675 meV) despite the specific *V*_O_ arrangement: The S0 polaron transfers part of the electronic charge into the molecular orbitals, thus stabilizing the adsorbing CO [see inset in Fig. 5a]. Figure 5b shows the corresponding simulated scanning tunneling microscopy (STM) images. The double-lobe signal of the CO molecule adsorbing on S0 polarons appears brighter compared to configurations without polarons, which is a clear indication of the sizable transfer of polaronic charge into the molecular orbitals. We note small differences in the orientation of the double lobes of the polaron-CO complexes and on ${E}_{{\rm{ads}}}^{F}$, which are due to the interaction with the oxygen vacancies in the proximity, lying in different arrangements.

The ${E}_{{\rm{ads}}}^{F}$ energy in Fig. 5a also allows us to clarify the contribution of Nb. As mentioned above, the ${E}_{{\rm{ads}}}^{GS}$ value obtained for a Nb lying on the S1 layer directly below the polaronic adsorption site is quite unfavorable (−383 meV). This is due to the high configuration energy of subsurface Nb atoms, which is significantly worse compared to that of the reference GS system used to calculate ${E}_{{\rm{ads}}}^{GS}$. Conversely, from ${E}_{{\rm{ads}}}^{F}$ we can assess the absence of any sizable role of Nb in the CO adsorption. In fact, the ${E}_{{\rm{ads}}}^{F}$ value for this case is in the typical range of polaron-CO complexes, despite Nb being in the proximity of the adsorption site (see also structural model in Fig. SF5 in the Supplementary Information). To further validate this conclusion, we modeled the CO adsorption on slabs without Nb defects. The results show that the ${E}_{{\rm{ads}}}^{F}$ adsorption energy is largely unaffected by the removal of Nb (going from −720 meV with Nb to −760 meV without Nb in the case of S0-polaron driven adsorption, and from −590 to −560 meV in the absence of surface polarons; see also Fig. SF6).

Therefore, these results further confirm that Nb does not play a direct role in CO adsorption, in contrast to the key contribution of oxygen vacancies. The ability to disentangle these complex defect-polaron interactions and clarify the role of individual dopants stems directly from the powerful and efficient framework developed in this study: the machine-learning acceleration and automated modeling allow us to move beyond simplified models and to treat multiple defects simultaneously. Compared to earlier works, our results confirm the key role of oxygen vacancies in stabilizing surface polarons interacting with adsorbates^22,23^, and at the same time rule out sizable effects from Nb, an aspect not assessed before. Our systematic exploration of the configurational space ensures that these conclusions are robust and not dependent on a limited set of configurations.

In summary, the computational package we introduced enables efficient and fully automated DFT modeling of small polarons in materials. It provides a powerful framework to uncover the fundamental properties of polarons and defects, and to gain insight into technologically relevant phenomena—such as surface reactivity, which we explored here for TiO_2_(110) as a case study. The MLFF-localization tool included in the package enables rapid and reliable trapping of polarons on user-defined sites. By leveraging data from finite-temperature DFT molecular dynamics and training the MLFF with polaron-specific labels, our method yields atomic structures with localized charges at target positions, while maintaining DFT accuracy at a fraction of the computational cost. The PolFlow high-throughput workflow streamlines and automates all steps required for polaron modeling. It coordinates polaron localization calculations, monitors localization success in real time, and triggers corrective measures when needed. This ensures robust convergence toward physically meaningful polaronic solutions with minimal manual intervention, making polaron modeling more accessible and reproducible. Additionally, PolFlow features an interface to active-learning frameworks for exploring the configurational space of defects and polarons. Using simulated annealing and ML-predicted energies, this module identifies low-energy configurations and iteratively improves its predictions by selecting new, informative ones for training the model.

We applied this package to Nb-doped TiO_2_(110) surfaces with oxygen vacancies to investigate how polaron-defect interactions affect surface reactivity. Our results show that oxygen vacancies play a central role in stabilizing surface polarons, especially in specific vacancy arrangements such as the 2 × c pattern. In contrast, Nb dopants contribute by donating excess electrons to the system, but have a limited impact on the stability of surface polarons.

Adsorption energy analysis, using CO as a probe molecule, revealed that surface polarons significantly enhance the adsorption strength on T*i*_5c_ sites by transferring charge to molecular orbitals. These polaron-CO complexes are practically unaffected by Nb dopants on the S2 layers, and are perturbed only to a minor extent by the local oxygen-vacancy arrangement: While *V*_O_ defects play a key role in stabilizing S0 surface polarons, they show minor effects on the polaron-CO complexes, such as slight rotations in the simulated STM signals and small variations in the adsorption energies.

These findings highlight the importance of efficient configuration sampling in first-principles simulations to reveal microscopic links between defect configurations, polaron distributions, and catalytic activity on oxide surfaces. The integration of machine-learning acceleration and automated workflows proves particularly valuable for overcoming the practical limitations of traditional approaches, which typically require simplified models (*e.g*., small cells or few impurities) and restrict the exploration of configurational space. Our work establishes a robust computational framework for modeling such defect-polaron interplay, opening new research paths for understanding and predicting the role of polarons in functional materials, with potential applications extending from catalysis to electronic and energy technologies.

## Methods

In this Section, we describe our automated approach to polaron modeling in the supercell-DFT framework. As sketched in Fig. 1, our software package features three components, addressing different tasks:

- an MLFF-powered localization technique to model polaron trapping on target sites, e.g., user-defined lattice sites (see Section “Localizing polarons via MLFF”);
- an efficient workflow (PolFlow) designed to streamline the series of DFT calculations required to obtain polaron trapping, including an automated on-the-fly control on the status of the simulations (see Section “Workflow to streamline polaron modeling”);
- an interface to an active learning scheme (ConfML) for exploring the configuration space of polarons and atomic defects in the system under investigation (see Section “Active learning for defect-polaron configurations”).

A description of the computational setup specifically adopted in our study case, i.e., defect-polaron configurations on the (110) surface of rutile TiO_2_ with oxygen vacancies and Nb doping, is provided in Section “Computational setup for the TiO_2_(110) study case”.

### Localizing polarons via MLFF

We propose a robust and efficient approach to model small-polaron localization based on ML force fields, as illustrated in Fig. 1a.

As a preliminary step of the MLFF-localization approach, the force field must be trained if not already available for the polaronic system under investigation. To this aim, short DFT-based molecular dynamics (DFT-MD) simulations using the on-the-fly learning scheme implemented in the Vienna Ab-initio Simulation Package (VASP)^88,89^ are used to efficiently generate a good training dataset. The finite temperature in DFT-MD simulations normally leads to spontaneous polaron trapping in polaronic systems, although with no control over the specific localization site. Thermally activated hopping typically causes the polaron to localize on different lattice sites during the DFT-MD. As a result, the DFT-MD trajectory naturally samples a variety of localized polaron configurations, enriching the training set and improving the quality of the force field.

The local change in atomic valence states caused by polaron localization poses challenges to standard MLFF techniques^90–92^. To overcome this limitation, we adapted the MLFF descriptors to account for polaron localization by labeling the trapping sites in the DFT-MD dataset as polaronic species, denoted “Ps”. This assignment is performed by identifying sizable perturbations of the local magnetic moments, compatible with polaron localization (e.g., magnetic moments larger than 0.5 μ_B_ in TiO_2_), and labeling the hosting atom as Ps. The DFT-MD dataset, with the polaronic atoms properly labeled, is used to train the kernel-based MLFF model implemented in VASP^88,93^ to evaluate forces and energies in the presence of polarons.

Once trained, the MLFF can be used to localize polarons at any site: by simply labeling as Ps the target sites designated to host the polarons, an MLFF calculation can be performed to obtain the charge-trapping atomic structure, as well as the corresponding energy and forces. The MLFF results can then be further refined through a standard DFT calculation.

This MLFF-based localization method is powerful and highly efficient. It provides users with a simple and convenient tool to select trapping sites, and the MLFF calculations are orders of magnitude faster than standard DFT, while maintaining DFT-level accuracy. A detailed description of the performance is given in Section “Benchmark and performance analysis”.

Finally, although this MLFF-based technique for polaron localization can be used independently, we have embedded it into our workflow to further facilitate its use (see the following section below for a description of the workflow).

### Workflow to streamline polaron modeling

In this Section, we present the automated workflow PolFlow, designed to streamline the process of polaron localization within supercell DFT simulations. Our goal is to make polaron modeling more accessible, reducing the knowledge barrier required to conduct in-depth investigations of polaronic materials. To this aim, the workflow is constructed to handle the entire process from input file preparation to calculation execution, and it automatically generates a database of results, which serves as a valuable resource for subsequent post-processing and analysis.

Figure 1b outlines the main tasks performed by the workflow, namely polaron localization calculations, checks on the charge trapping status, and, eventually, exploration of the configuration space. To perform these tasks, it leverages a suite of software tools, including Fireworks^94^ as a workflow manager (which automates and coordinates the execution of complex computational tasks), atomate^95^ (an interface for VASP-specific workflows), pymatgen^96^ for structure generation and manipulation, and custodian^96^ for on-the-fly error checking and corrections.

As shown in Fig. 1b, the workflow is initialized using a user-defined structure (optionally obtained from the Materials Project^97^ database via pymatgen) and a set of indices for the positions of defects and polarons in the system. It then automatically sets up the DFT calculations required to localize the polarons at the specified sites. If a pre-trained MLFF model is available (see Section “Localizing polarons via MLFF”), the workflow performs an MLFF relaxation of the atomic coordinates, with the polaronic species Ps at the custom positions. A DFT calculation then follows, starting from the relaxed structure as obtained via the MLFF (referred to in the following as MLFF+DFT strategy). During the execution of the DFT calculation, the workflow checks for possible localization issues: in the case one of the polarons becomes delocalized or re-localizes on a different site, the calculation is interrupted, and alternative methods to obtain polaron localization can be attempted. Specifically, we implemented an error-checking mechanism that monitors the status of polarons during runtime. This mechanism can dynamically initiate new runs or terminate the entire workflow if polaron migration or delocalization is detected, allowing adaptive responses to these issues and saving a significant amount of computational time (see Section “Results” for our analysis on the performance).

In the current implementation of the workflow, if a failure of polaron localization is detected after the MLFF+DFT step, a second localization attempt is performed using the occupation matrix control tool (OCCMAT)^43^. In this approach, the electronic occupation of the orbitals of the site designed to host the polaron is explicitly constrained to a polaronic solution, followed by a structural relaxation. The resulting structure and wavefunction are then used as input for a standard, unconstrained DFT relaxation (referred to in the following as OCCMAT+DFT).

If the localization is successful, all relevant information is stored (e.g., the free energy for the given defect-polaron configuration). The workflow is also designed to progressively increase the accuracy level of the DFT calculations. In practice, the localization attempts are initially performed using a faster, lower-accuracy setup; once a localized polaronic solution is obtained, the outputs (such as nuclei coordinates, electronic density, etc.) are used to run calculations at higher accuracy (e.g., finer sampling of the reciprocal space, higher energy cutoff, tighter convergence thresholds, etc.). This progressive refinement ensures accuracy while saving a considerable amount of computational time.

The workflow can be executed repeatedly in order to investigate the configuration space, inspecting different arrangements of polarons and defects. An efficient sampling can be obtained by interfacing the workflow with recently developed ad-hoc tools for accelerating the exploration of defect-polaron configurations^20,54,98^, as described in the following section.

### Active learning for defect-polaron configurations

The efficient automation of polaron localization via PolFlow facilitates systematic exploration of the defect-polaron configuration space. To accelerate this, we interfaced the workflow with a machine learning model (ConfML) that can be trained to predict the energy of any defect-polaron configuration, based on DFT data^20^, sketched in Fig. 1c. The model, implemented using the JAX framework^99^, consists of polaron- and defect-type-specific feed-forward neural networks, optimized via stochastic gradient descent and backpropagation.

We design the PolFlow workflow to be able to automatically define unexplored and symmetrically inequivalent defect-polaron arrangements, run the localization calculations, and compute the configuration energies required to train the ConfML model, thus implementing an active learning scheme. During the exploration, we use simulated annealing to optimize defect-polaron configurations, seeking to identify the ground-state structures, in line with our previous work^20^. Starting from a given or random configuration, defects and/or polarons are transferred to different sites. ML energies are then used to determine the Metropolis acceptance probability of the new configuration, which governs whether the configuration change is accepted or rejected using a probabilistic method. This approach allows for efficient exploration of the configuration space. Comparisons between ML predictions and DFT data are automatically performed in order to identify outliers and incorporate them into the training set to improve model accuracy.

To focus on efficiently exploring low-energy configurations rather than searching the entire polaronic configuration space, we propose a multi-phase approach. In the first phase, we generate a diverse initial dataset by randomly sampling the defect and polaronic configuration space. This ensures a broad coverage of the possible configurations, providing a solid foundation for our ConfML model. The random sampling is intended to avoid biases in the training. In the second phase, the ConfML training dataset is expanded with configurations obtained through simulated annealing, together with additional random polaron configurations in low-energy defect arrangements. Finally, in the third phase, we focus on sampling the low-energy subspace as predicted by the annealing. This targeted approach allows us to refine our understanding of the most stable and relevant polaron and defect arrangements. The low-energy ML predictions are evaluated against explicit DFT calculations of the proposed polaron configurations.

Throughout this active learning process, we iteratively improve reliability and efficiency, thus determining the ground-state and low-energy defect-polaron configurations.

### Computational setup for the TiO_2_(110) study case

This Section describes the computational setup adopted for our investigations on the rutile TiO_2_(110) surface with Nb dopants and O vacancies.

We performed DFT calculations using VASP^100,101^. We adopted the generalized gradient approximation (GGA) within the Perdew, Burke, and Ernzerhof (PBE) parametrization^102^, with the inclusion of an on-site effective *U*^103^ of 3.9 eV on the *d* orbitals of Ti and Nb atoms (this setup has been reported to agree with experiments concerning the distribution of defects on rutile surfaces^20^). Brillouin-zone sampling was restricted to the *Γ*-point for all calculations. In the MLFF training, the (OCCMAT) constrained calculations, as well as the low-accuracy DFT relaxations, a plane-wave energy cutoff of 250 eV was used. The cutoff was increased to 400 eV in the final calculations (referred to as high-accuracy DFT calculations). To train the MLFF, we performed short (15 ps) MD simulations in the NVT ensemble, starting from five different random defect-polaron configurations, as well as the corresponding delocalized configurations. The time step was set to 1 fs and the temperature to 300 K. Ti and Nb atoms showing a local magnetic moment larger than 0.5*μ*_B_ were labeled as distinct polaronic species “Ps" (in fact, the non-zero local magnetic moment is a fingerprint of electron polaron localization in *d*^0^ transition metal oxides^39^). In total, 202 training structures were collected. We used a radial cutoff of 8 and 6 Å for the two- and three-body descriptors, respectively, and 12 and 8 radial basis functions^93^. The training errors were 0.8 meV/atom for the energy and 0.08 eV/Å for the forces. The ConfML model for the exploration of the configuration space uses vanilla stochastic gradient descent with a learning rate of 0.005^20^.

Calculations on the adsorption of CO molecules included van der Waals interactions using the non-local SCAN-rVV10 functional^104–106^. The spatial extension of the electronic density and STM images simulated in the Tersoff-Hamann approximation^107^ were calculated by selecting an energy range corresponding to the polaronic eigenvalues only.

Our surface model, shown in Fig. 6, consists of a 6 × 3 slab with six TiO_2_ layers (the two bottom stoichiometric layers fixed in their bulk positions). To simulate the effects of thermal expansion observed during experimental annealing, we used an expanded [001] lattice vector with a constant of 2.968 Å, corresponding to the high-temperature regime^108,109^. We indicate with A the five-fold coordinated Ti atoms (T*i*_5c_) on the S0 surface layer and the Ti below, on S1 and S2 layers; B denotes instead all other Ti atoms on S0–S2 layers (see also labels in Fig. 6).

The slabs contain two surface oxygen vacancies (yielding a vacancy concentration of 11%, a typical value for 1 × 1 terminations of annealed rutile samples used to study chemical reactivity)^21,22,49,67^ and an Nb atom at a Ti site on the subsurface layers S1 and S2 (resulting in a dopant concentration of approximately 0.5%)^60^. These defects produce five excess electrons that can form polarons (four from the oxygen vacancies and one from the Nb dopant). Our initial dataset comprises 198 symmetrically distinct configurations. The A sites on S0, and all the Ti sites on S1 and S2 are considered as possible sites for polaron localization.

## Supplementary information

Supplementary Information

## Data Availability

The datasets generated and analyzed during this study are openly available in the Zenodo repository, see ref. ^110^.

## Code availability

The code developed for this study is openly available in the Zenodo repository, see ref. ^110^. The Zenodo record points to the GitHub repository where the code is hosted.
