# Supplementary material for: Automated modeling of polarons: defects and reactivity on TiO2(110) surfaces
Source: NPJ Comput Mater. 2026 Apr 9;12(1):254. doi: 10.1038/s41524-026-01983-5 (PMC13427633; doi:10.1038/s41524-026-01983-5)
Supplement: Supplementary file 1 — Supplementary Information [file 41524_2026_1983_MOESM1_ESM.pdf]

Supplementary Information: Automated Modeling of Polarons: Defects and Reactivity on TiO_2(110) Surfaces

Firat Yalcin,[1,*] Carla Verdi,[2,†] Viktor Birschitzky,[1] Matthias Meier,[1] Michael Wolloch,[1,3] and Michele Reticcioli[1,4,5,‡]

[1] *University of Vienna, Faculty of Physics and Center for Computational Materials Science, Kolingasse 14-16, 1090 Vienna, Austria*
[2] *School of Mathematics and Physics, The University of Queensland, Brisbane 4072 QLD, Australia*
[3] *VASP Software GmbH, Berggasse 21/14, 1090 Vienna, Austria*
[4] *National Research Council, CNR-SPIN, via Vetoio 42, 67100 L'Aquila, Italy*
[5] *University of L'Aquila, via Vetoio 10, 67100 L'Aquila, Italy*

CONTENTS

I. Supplementary Information on the Machine-Learning-Model Performance 2
II. Site-Projected Density of States 4
III. Effect of Polaron-Polaron Interactions 5
IV. Nb Dopant on S1 Subsurface Layer 6
V. CO Adsorption on Nb-free Surfaces 7
References 8

I. SUPPLEMENTARY INFORMATION ON THE MACHINE-LEARNING-MODEL PERFORMANCE

The active-learning approach implemented in the `PolFlow` package[1] enabled efficient training of the ConfML model[2]. The methodology is described in the main text. Here we present a complementary analysis of the model's prediction accuracy across both the entire energy range and the low-energy region (of our database including 1924 symmetrically distinct configurations, as described in the main text). As demonstrated in Figure SF1, our machine learning model can

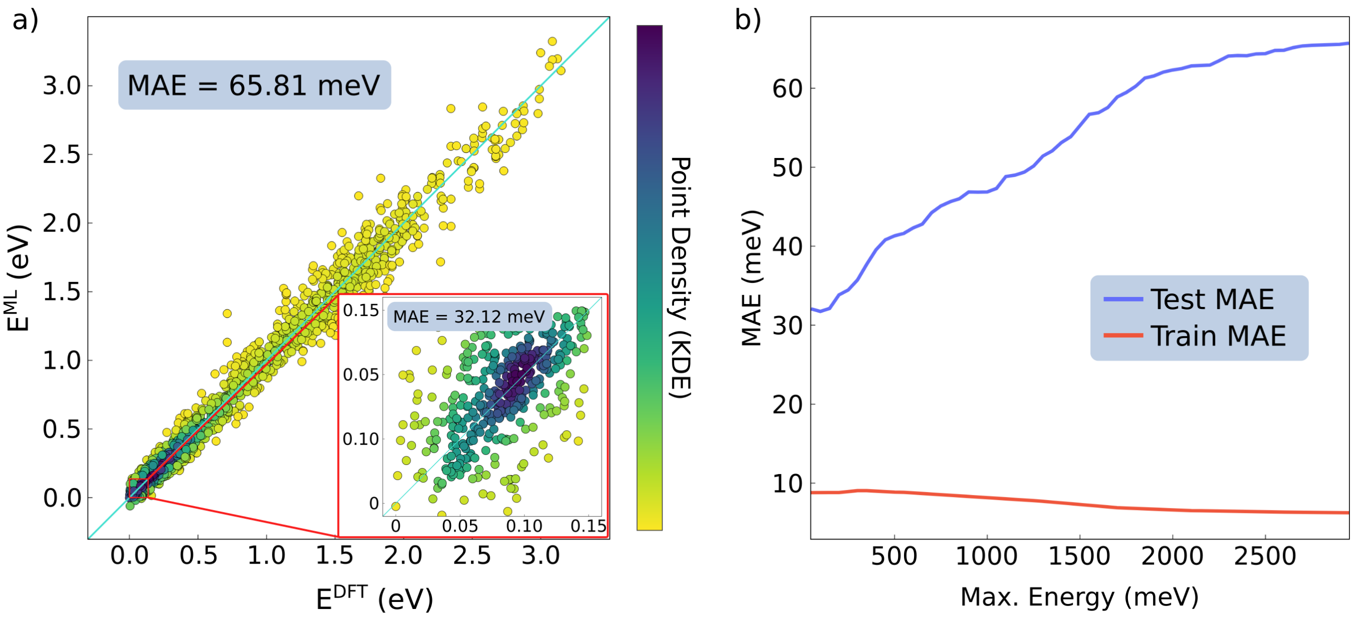

FIG. SF1. **Predictive accuracy and energy-dependent error analysis of the ConfML model compared to DFT calculations.** **(a)** Scatter plot comparing predicted energy values from the ConfML model to DFT energy values for training and test datasets. The plot shows an overall MAE of 65.81 meV for the full dataset, corresponding to approximately 13 meV per polaron. The red rectangle highlights a smaller energy range (up to 0.15 eV), where the model achieves improved accuracy with a MAE of 32.12 meV (6 meV per polaron). **(b)** Mean absolute error for the test and train datasets plotted over maximum energy threshold.

predict the energy of any defect-polaron configuration on $TiO_2(110)$ with a mean absolute error (MAE) of 65.81 meV for our 6×3 slab, corresponding to approximately 13 meV per polaron. Notably, the model's accuracy is significantly enhanced in the low-energy region that is most relevant for low-temperature physical properties. By considering only configurations with energies up to 0.15 eV above the ground state (highlighted in the red rectangle), the prediction error is reduced to 32.12 meV for the entire slab, or just 6 meV per polaron. As shown in panel b, the machine-learning test phase is highly sensitive to the energy range selected for the MAE analysis. This improved accuracy at low energies is a direct result of our multi-phase active learning approach. The annealing-based training in Phase 3, as shown in Figure 3 of the main text, specifically prioritized the most stable defect-polaron configurations, allowing the model to learn the subtle energy differences between closely competing arrangements. This targeted training strategy ensures that the model performs best precisely where accuracy matters most: near the ground state configuration.

Figure SF2 complements Figure 2(c) in the main text. Here, we show the overall runtime comparison between MLFF+DFT and OCCMAT+DFT. In our system, the overall polaron localization process via the OCCMAT+DFT approach required on average 1.5 times the computing time compared to the MLFF+DFT strategy per defect-polaron configuration (corresponding to 7 and 4.7 hours, respectively). Obviously, the gain of the MLFF-powered strategy is more evident in the comparison of the low-accuracy calculations (3 times faster, as described in the main text): This is due to having trained the MLFF routine on a molecular dynamics simulation using the low-accuracy setup.

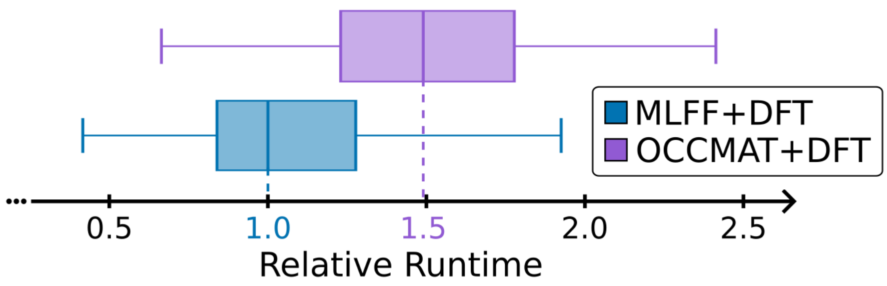

FIG. SF2. **Runtime of the MLFF+DFT and OCCMAT approaches in the full (low- and high-accuracy) runs.** Boxplots show the distribution of computational times normalized to the MLFF+DFT median. The corresponding analysis for the low-accuracy set of calculations is shown in Fig. 2c in the main text.

II. SITE-PROJECTED DENSITY OF STATES

In the discussion of the electronic structure in the main text (specifically related to Figure 4), we focused on the density of states (DOS) projected onto the surface (S0) polaron and subsurface (S1) polarons either isolated or localizing three lattice sites away from a second polaron along the same [001] Ti row. This was done to highlight the shift of the S0 polaron eigenstate and the broadening of the subsurface polaronic peaks due to repulsive interactions between in-row subsurface polarons. However, to provide a complete picture of the electronic structure and clarify the contribution of all polaronic species, we present here the DOS projected onto the orbitals of all Ti atoms hosting polarons.

Figure SF3 shows the spatial distribution (top panels) and the corresponding electronic states (bottom panels) for the $0 \times c$, $1 \times c$, and $2 \times c$ configurations. The DOS are projected on the Ti atoms hosting polarons, with different colors distinguishing the specific localization sites.

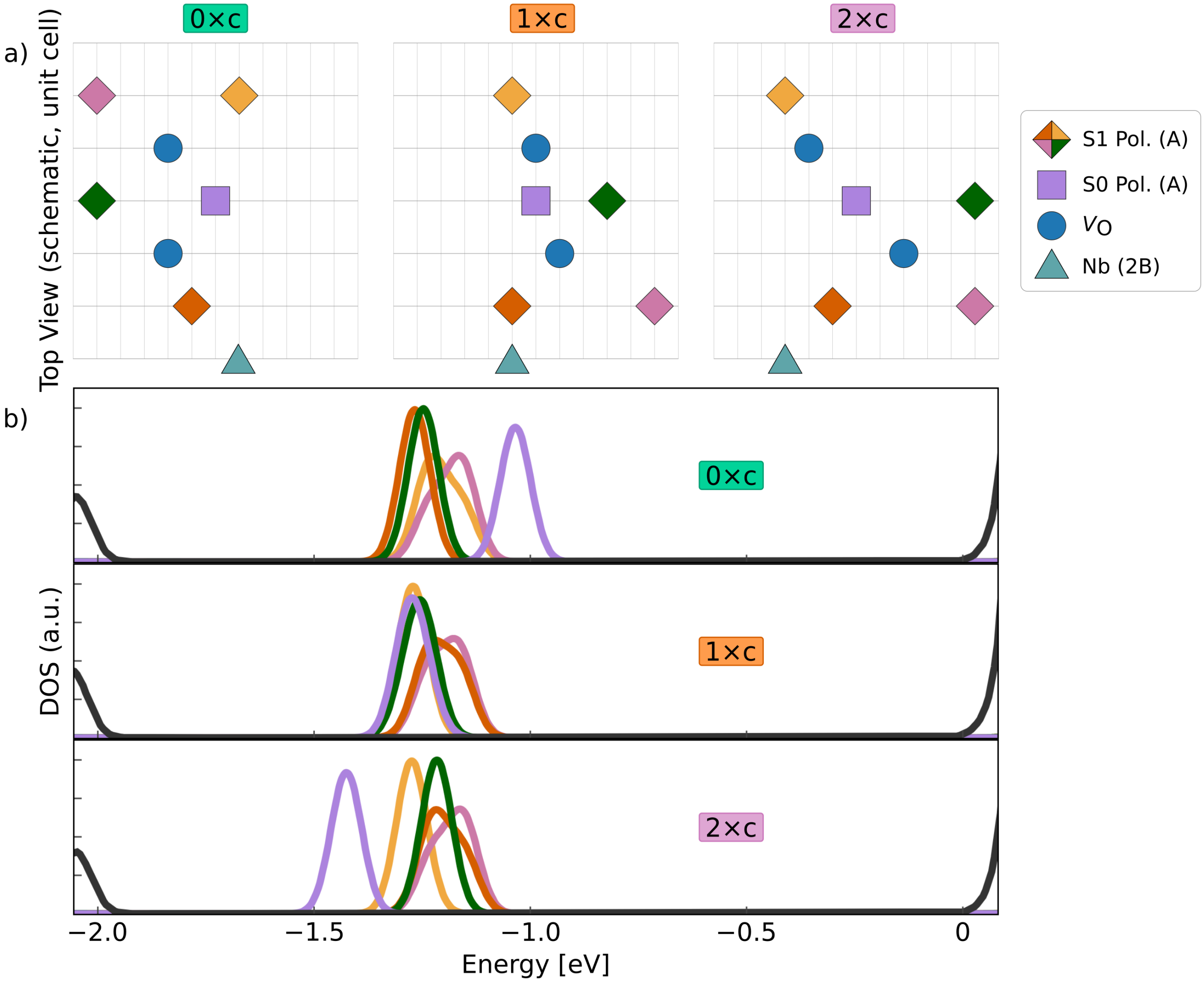

FIG. SF3. **Projected density of states (DOS) and spatial configuration of defects for the $0 \times c$, $1 \times c$, and $2 \times c$ charge states. Top panels:** Spatial arrangement of the defects relative to the surface lattice. The symbols indicate oxygen vacancies (V_O, blue circles), Nb dopants (teal triangles), surface polarons (S0, purple squares), and subsurface polarons (S1, various colors). **Bottom panels:** The corresponding DOS projected onto all Ti atoms hosting polarons. The colors of the DOS curves match the symbols of the localization sites in the top panels. This figure complements Figure 4 in the main text by including projections for all polaronic sites, rather than showing only the S0 contribution.

III. EFFECT OF POLARON-POLARON INTERACTIONS

Our extensive sampling of the defect-polaron configuration space allows us to investigate not only defect-polaron interactions but also the effects of polaron-polaron interactions on the electronic structure. As mentioned in the main text, the repulsive interaction between polarons can significantly impact their stability and, under highly reducing conditions, even trigger surface reconstructions.

Figure SF4 illustrates this effect for our ground-state 2×c configuration. In this arrangement, multiple polarons are localized on the S1 subsurface layer. As shown in the structural model in Fig. SF4(a), two of these S1 polarons are positioned along the same [001] Ti row, separated by three lattice sites. The projected density of states (DOS) in Fig. SF4(b), which is also presented in the main text, reveals the electronic consequence of this proximity. The DOS peak for the 'in-row' S1 polaron (blue curve) is visibly broadened compared to the sharper peak of a more isolated S1 polaron within the same supercell (gray curve). This broadening is a characteristic signature of the repulsive interaction between the two nearby polarons, which destabilizes their energy levels. This finding corroborates previous studies and highlights the ability of our automated workflow to capture subtle but crucial electronic effects arising from the complex interplay of defects and charge carriers.

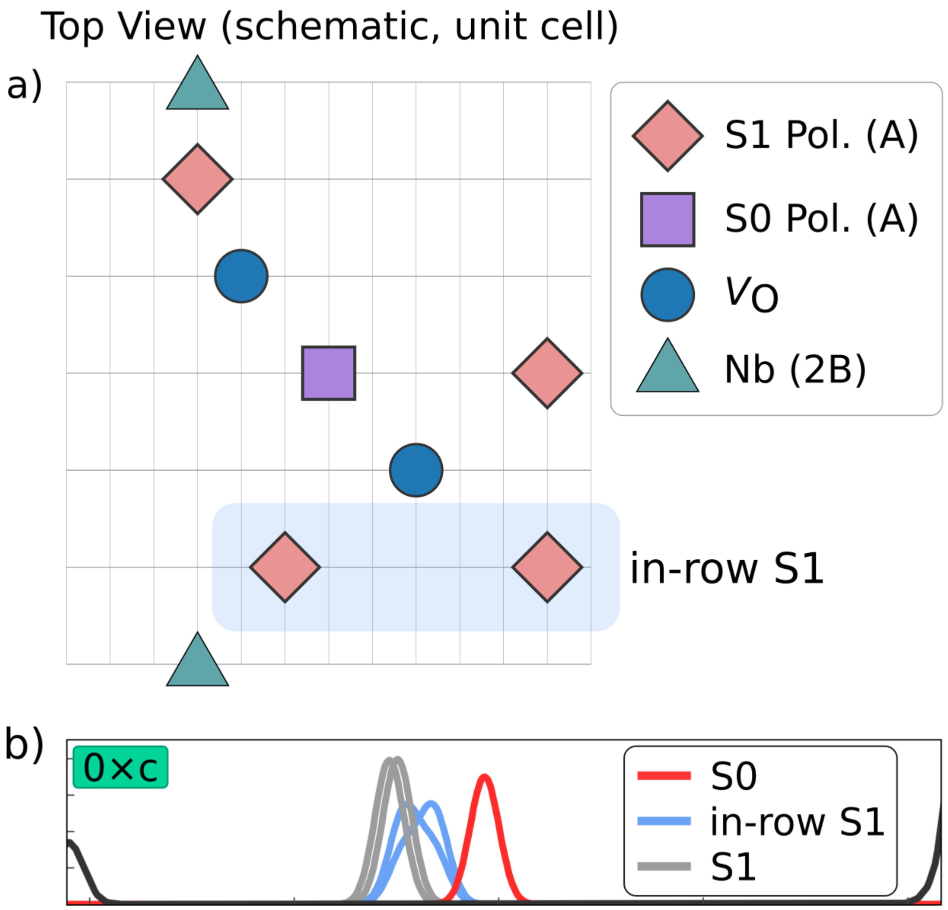

FIG. SF4. **Analysis of polaron-polaron interactions in the ground-state 2×c configuration. (a)** Top schematic view of the atomic structure, highlighting the oxygen vacancies (V_O) and the polaronic sites. Two polarons on the S1 layer are aligned along the same [001] Ti row ('in-row'). **(b)** Projected density of states (DOS) showing the sharp eigenstates of isolated S1 polarons (gray) compared to the broadened eigenstates of the in-row S1 polarons (blue), indicating repulsive polaron-polaron interactions. The surface S0 polaron state is shown in red for reference.

IV. NB DOPANT ON S1 SUBSURFACE LAYER

Our configuration space exploration revealed a clear preference for Nb dopants to occupy sites on the S2 layer rather than positions on the S1 layer. To understand the electronic origin of this preference and its implications for surface reactivity, we can analyze the specific configuration shown in Figure SF5, which features an Nb dopant on the S1 subsurface layer. This structure, which corresponds to the configuration represented by a square symbol in Figure 5 of the main text, is particularly informative for understanding both the instability of S1 Nb dopants and their direct influence on CO adsorption.

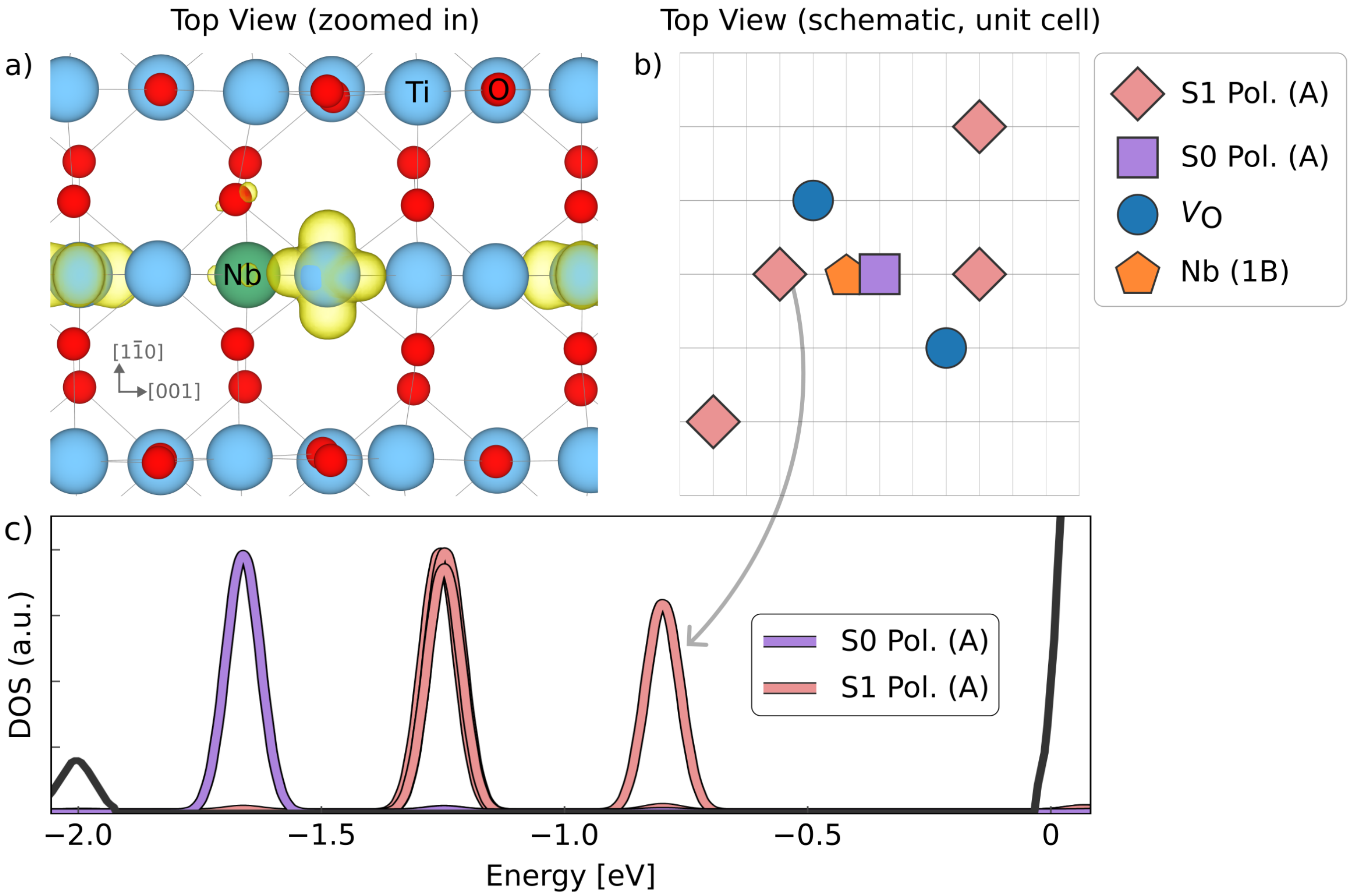

FIG. SF5. **Analysis of a configuration with an Nb dopant on the S1 subsurface layer.** **(a)** Top-down, zoomed-in view of the relaxed atomic structure, showing the Nb atom and a nearby S1 polaron (yellow isosurface). **(b)** Schematic representation of the defect positions within the unit cell. **(c)** Projected density of states (DOS) comparing the S0 and S1 polarons. The DOS reveals that the S1 polaron in proximity to the S1 Nb dopant has a significantly higher energy eigenvalue compared to the S0 polaron, explaining the energetic instability of this configuration.

The projected density of states (DOS) in Figure SF5c provides a clear electronic fingerprint for the instability of this configuration. The eigenvalue for the S1 polaron located near the dopant is exceptionally high, indicating a destabilization of the polaronic state. This unfavorable electronic structure likely stems from the local distortion of the atomic structure around the S1 Nb dopant, which creates a less suitable environment for polaron trapping compared to the more stable S2 configurations.

This same structural model is also useful for evaluating the direct role of Nb in surface reactions. The Nb dopant is in close proximity to the surface, positioned directly below a potential CO adsorption site. Despite this, our E^F_{ads} (as defined in Eqn. 3 of the main text) analysis showed that the dopant has a minimal direct effect on the adsorption strength of CO. The reason this configuration is unfavorable for the overall adsorption process (as measured by E^{GS}_{ads}) is not because of a weak interaction with CO, but because of the high intrinsic energy of having an Nb atom at an unstable S1 site. This observation provides strong evidence that the primary role of Nb dopants is to donate excess electrons to the system, rather than to directly participate in surface chemical reactions.

V. CO ADSORPTION ON NB-FREE SURFACES

To further isolate and quantify the effect of Nb dopants on surface reactivity, we performed additional calculations on Nb-free surfaces and compared the resulting adsorption energies with those obtained for Nb-doped systems. Figure SF6

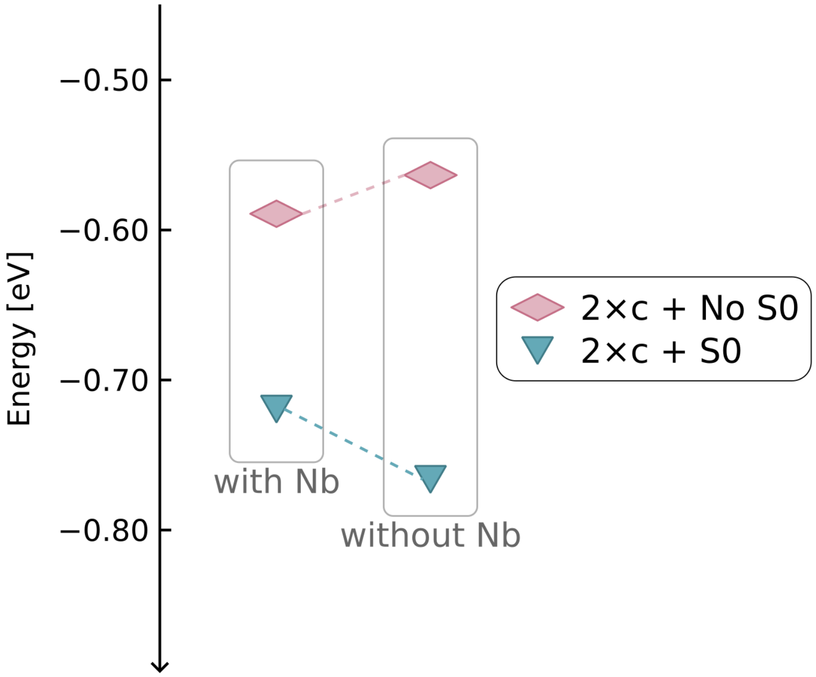

FIG. SF6. **Comparison of $E^F_{\rm ads}$ adsorption energies for CO on TiO_2(110) surfaces with and without Nb dopants.** The figure shows similar adsorption energies in both cases: for S0-polaron driven adsorption, the energy changes from −720 meV with Nb to −760 meV without Nb, while for surfaces without S0 polarons, it changes from −590 meV to −560 meV. These results confirm the minimal direct impact of Nb dopants on CO adsorption.

compares the $E^F_{\rm ads}$ adsorption energies for CO on TiO_2(110) surfaces with and without Nb dopants. The data clearly demonstrates that removing the Nb dopant has minimal effect on the CO adsorption energy. For configurations with S0 polarons, the adsorption energy changes from −720 meV with Nb to −760 meV without Nb—a relatively small difference of 40 meV. Similarly, for configurations without surface polarons, the adsorption energy changes from −590 meV to −560 meV—an even smaller difference of 30 meV in the opposite direction.

These results provide strong evidence that Nb dopants play a negligible direct role in CO adsorption on TiO_2(110) surfaces. This finding aligns with our overall conclusion that while Nb doping is effective for introducing additional electrons into the system, it does not significantly alter the surface reactivity toward CO molecules. Instead, oxygen vacancies remain the dominant factor in determining surface reactivity, primarily through their role in stabilizing polarons on the surface atomic layer, which then serve as active centers for molecular adsorption.

* firat.yalcin@univie.ac.at
† c.verdi@uq.edu.au
‡ michele.reticcioli@univie.ac.at

[1] F. Yalcin, "polflow: v1.0.0 Initial Public Release," (2025).
[2] V. C. Birschitzky, I. Sokolović, M. Prezzi, K. Palotás, M. Setvín, U. Diebold, M. Reticcioli, and C. Franchini, *"Machine learning-based prediction of polaron-vacancy patterns on the TiO2(110) surface,"* npj Computational Materials 10, 89 (2024).
